# Cell Density Detector Based on Light Beam Focusing

**DOI:** 10.3390/mi9110592

**Published:** 2018-11-13

**Authors:** Aoqun Jian, Huiming Li, Yixia Zhang, Qianqian Duan, Qianwu Zhang, Shengbo Sang

**Affiliations:** 1 MicroNano System Research Center, College of Information Engineering and Computer Science, Taiyuan University of Technology, Jinzhong 030600, China; jianaoqun@tyut.edu.cn (A.J.); lihuiming_0406@sina.com (H.L.); wwhwls@163.com (Q.D.); 2 Key Laboratory of Advanced Transducers and Intelligent Control System, Shanxi Province and Ministry of Education, Taiyuan 030024, China; 3 Institute of Applied Mechanics and Biomedical Engineering & National Demonstration Center for Experimental Mechanics Education, College of Mechanics, Taiyuan University of Technology, Taiyuan 030024, China; zhangyixia@tuyt.edu.cn; 4 Key Lab Specialty Fiber Opt & Opt Access Networks, Shanghai University, Shanghai 200072, China; qianwuz@gmail.com

**Keywords:** cell counting, microfluidics system, light beam focusing

## Abstract

Although the lab-on-a-chip system has been successfully applied in a wide variety of fields, the goal of achieving a cell counter with simple operation, low cost, and high accuracy still attracts continuous research efforts. In this paper, the authors explore a cell counter based on light beam focusing to measure the density of adherent cells. In this sensor, the light emitted from the optical fibers is collimated by the collimating lens formed in polydimethylsiloxane (PDMS). The uniformly attached adherent cells act as a convex lens, focusing the collimated light propagated through them. The intensity of the focused light indicates the density of the adherent cells. For Hela cells, a detection limit of 8.3 × 10^4^ cells/mL with a detection range from 0.1 × 10^6^ cells/mL to 1.0 × 10^6^ cells/mL is achieved. This sensor is particularly useful for drug screening, cell pathology analysis, and cancer pre-diagnosis.

## 1. Introduction

The cell is the main element of the human body, as well as the basic unit of human life activities. Abnormality in the number of cells is an indicator for a disease state of the human body. The pathological relationship between retinal detachment and reduction can be diagnosed by analyzing retinal cell density [1]. A patient’s pathophysiology can be determined by blood cell density [2]. In the microbiology industry, the accurate counting of yeast cells can more accurately determine the density and activity of yeast cells, which will facilitate subsequent fermentation studies [3]. In addition, during the outbreak of red tides in the coastal areas of China, the determination of algae cell density and species in the sea directly affected the strategic decisions made by the government. To summarize, the accurate detection of cell numbers is of great significance for disease diagnosis, drug screening, and environmental pollution monitoring.

At present, the methods of cell counting widely used can be classified into three types: cell counting plates, Coulter counters, and flow cytometers [4]. The cell counting plate is achieved by manually injecting the cell suspension into a counting plate and counting the number of cells under a microscope [5,6,7]. This method is used by most laboratories and scientific research institutions—from the first instances of cell experimentation to present day—due to its advantageously fast and easy operation [8]. However, this method has some insurmountable defects in the counting process such as low efficiency and large subjective errors [9].

The Coulter counter, discovered by Dr. W.H. Coulter in 1949 and patented in 1953 [10], is based on the principle that the cell passes through a small hole and affects the located resistance characteristics due to the difference in conductivity between the cell and the suspended medium, which form an electrical pulse signal at the same time. The size and number of the cells can be obtained by measuring the intensity and the number of electrical pulses [11,12,13]. As this method counts the cells one by one, it has the advantages of high accuracy and high throughput [14]. However, in the case of cells with high density, in order to complete the cell counting quickly, the cell transporting pipes must be increased (up to 512 channels) and the channel diameter must be larger than a certain size to avoid thermal noise, which makes the detection system complicated and bulky. In addition, the long-term use of the detection system also has problems such as the blockage of transport channels [15].

Flow cytometry (FCM) is a biomedical analysis technology that has undergone rapid development [16,17]. Its basic principle is based on light scattering: as the light emitted by the light source is focused and interacts with a single cell, the relevant cell information is acquired by detecting the spectrum of scattered light [18,19,20]. This method can realize the multi-parameter and rapid quantitative analysis of single cells or other biological particles with the advantages of high speed and high precision. However, the application of flow cytometry is limited by its high cost (about 200,000 dollars). Meanwhile, cells need to be stained, which is expensive and has a certain influence on the physiological activities of cells.

Based on the survey of cell counting devices/instruments made above, there is still a lack of cost-effective test instruments which can achieve the label-free (no staining) and accurate measurement of cell density. In this paper, a cell density detector based on light beam focusing is proposed, which can realize the measurement of cell density of adherent cells. The highly integrated device is prepared by the well-established MEMS technology [21,22,23], and its cost can be as low as 15.6 dollars. Without extra cell treatment and microscopic observation, a range of densities between 0.1 × 10^6^ cells/mL and 1 × 10^6^ cells/mL can be measured. Also, a detection limit (LOD) of 8.3 × 10^4^ cells/mL can be achieved by a simple operation.

## 2. Optical Sensors Design

### 2.1. Initial Motive and Design

As indicated by its name, the growth of adhered cells needs to be attached to a smooth surface [24,25]. When suspended adhered cells are statically cultured for a while, they tend to attach to the smooth surface (e.g., the substrate of the petri dish), collocate closely, and pave the container’s surface as a “cell layer”. This self-given natural characteristic of adhered cells offers the initial motive of our sensor.

**Figure 1:**
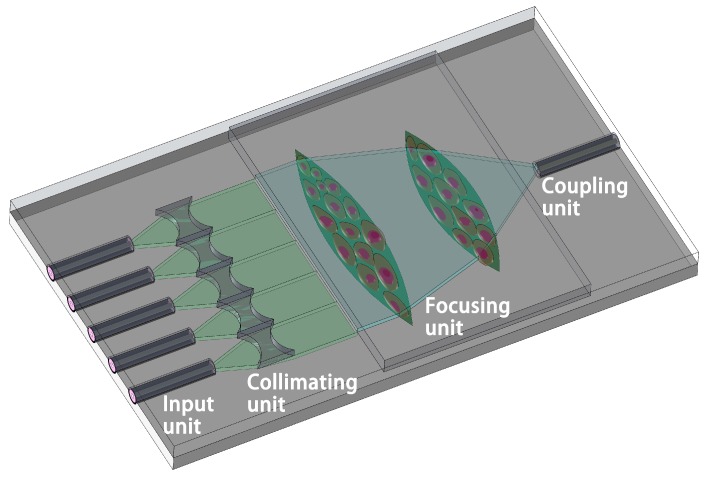
System schematic graph of the cell density detector.

The schematic graph of the cell density detector is shown in Figure 1. The device mainly includes four parts through which the light propagates: the input unit, the collimating unit, the focusing unit, and the coupling unit. Firstly, the divergent light emitted from the optical fibers is collimated by the collimating unit. The collimated parallel light is converged by the focusing unit, which is composed of adherent cells with different densities. The refractive index (RI) of the focus lens changes as the cell density is altered. Furthermore, the light intensities will be tuned due to the variation of the RI of the lens if the receiving fiber is fixed at a specific location. Thus, cell counting can be realized by detecting the intensity of the focused light.

The areas of the lens (focusing sections) are about 3.2 mm^2^ and 2.5 mm^2^, and the diameter of the cells are normally 10–20 μm (area 1.26 × 10^−3^ mm^2^−3.1 × 10^−4^ mm^2^). Therefore, thousands of cells will be localized in the focusing area, and their distribution is considered to be homogeneous. That is to say, the cells will randomly and uniformly cover the lens area due to the growth characteristics of adherent cells. Thus, the RI of the lens completely depends on the cell density attached to the lens area.

The critical components of the designed sensor (the input unit, the collimating unit, the focusing unit, and the coupling unit) are introduced in sequence in the following section of the paper.

### 2.2. Input Unit and Collimating Unit

The input unit, which is made up of five cuboid grooves arranged in parallel, is designed to fix the optical fibers and constrain the direction of input light propagation. As the most popular type of optical waveguide, optical fibers are utilized to transmit light in the device. Its compact size will give the device high integration abilities and the well-developed optical fiber-based components serve to cut the cost of the device. In this study, five multimode fibers with a cladding/core radius of 62.5/125 μm and a numerical aperture of 0.275 are used as the input light source.

In order to ensure that the optical fibers are accurately and firmly located in the cuboid groove, the length and width of the grooves are set as 20 mm and 260 μm, respectively. To achieve a 2-mm-wide parallel light beam in the experiment, the spacing of the groove elements is set as 400 μm. In addition, because the diameter of most cells is about 7–15 μm, the optical fiber is embedded in the cuboid groove, leaving only half of the inner core of the optical fiber outside.

**Figure 2:**
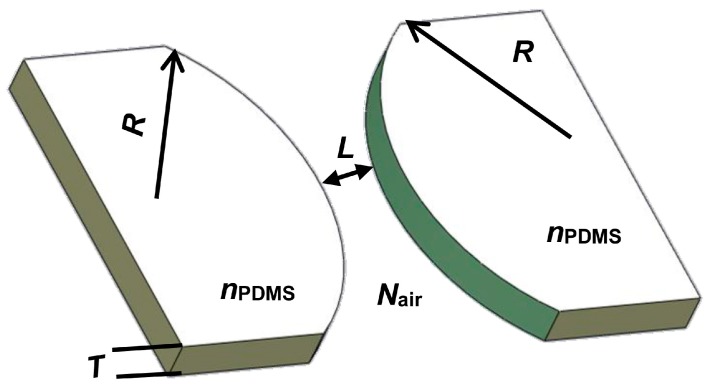
Device structure design of the collimating unit.

**Figure 3:**
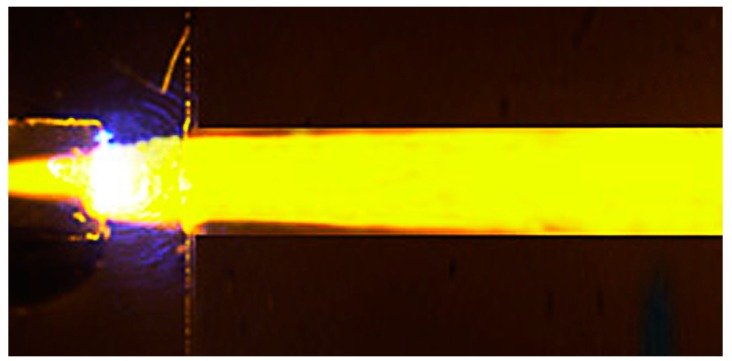
The experimental result of light passing through the collimating unit.

The collimating unit is made up of five collimating lenses integrated into polydimethylsiloxane (PDMS). The collimating lens model is presented in Figure 2. The dimensions of the collimating lens were obtained through a simple calculation of the transfer matrix method (TMM) by Mathematica [26]. The geometric dimensions of the collimating lens obtained are listed in Table 1. Figure 3 offers the experimental results of light passing through the collimating unit, which demonstrates the parallel propagating light achieved by the collimating unit successfully. The collimated light is traced by fluorescence dye (Rhodamine B).

### 2.3. Focusing Unit

The focusing unit is designed to focus the parallel beam emitted from the collimating unit, which is made up of focusing lenses composed of adherent cells. In the case of sensor design, a uniform high RI medium is adopted to mimic the effect of the assemblage of adherent cells.

**Figure 4:**
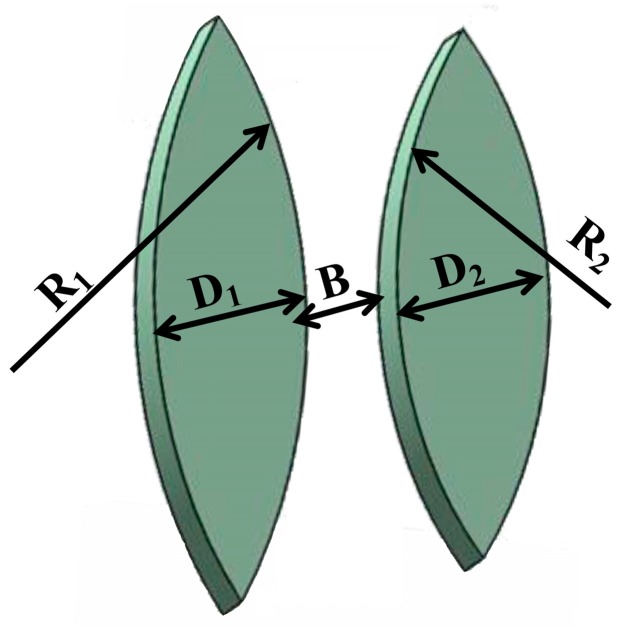
Device structure design of the focusing unit.

Figure 4 describes the model of the focusing lens. In order to enhance the focusing effect, a set of cascaded lenses are utilized in the system. Their structural dimensions (listed in Table 2) were obtained based on the computational simulation of Mathematica and Zemax; in this case, the thickness was found to be nearly equal to the diameter of the cell (7–15 μm). When adherent cells are statically cultured, they tend to attach to a smooth surface. Moreover, they will not overlap with each other but form a single cell-layer covering the bottom of the container. In this device, all the input and coupling optical fibers are embedded in the cuboid groove, leaving only half of the core of the optical fiber outside (about 30 μm). Although some parallel beams will directly pass through the focusing lenses, the light intensity collected by the coupling fiber comes mainly from the focused beams due to the small receiving aperture of the coupling fiber.

**Figure 5:**
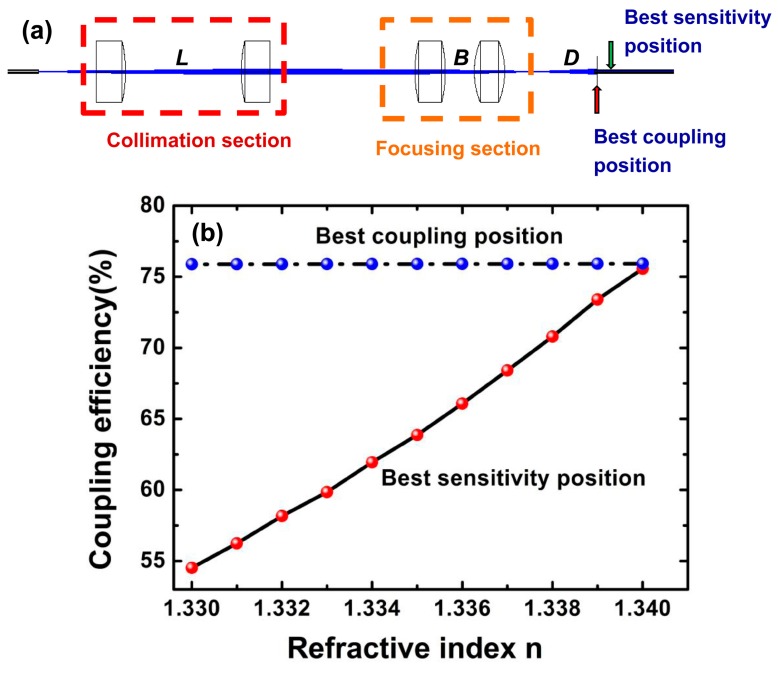
The coupling efficiency of the optical sensor for different RIs in two different positions: (**a**) the modeling diagram of Zemax; (**b**) simulation results.

The location of the coupling optical fiber was achieved using Zemax (shown in Figure 5a). Since Zemax does not support the close arrangement of multiple fibers, the light beam originally emitting from the fibers array was formed by a single multimode fiber and assorted collimating lenses in the simulation. It was found that when the distance (*D*) between the coupling fiber and the cascaded lenses is 2.7 mm, the fiber coupling efficiency is the highest, but it is not sensitive to the variation of the RI of cascaded lenses. Meanwhile, if the distance (*D*) is 3.1 mm, the coupling efficiency is more sensitive to the variation of the RI. Figure 5b describes the dependence of the coupling efficiency on the change of the RI in the two states with different *D* values. When the RI of the focusing lens varied from 1.330 to 1.340, the coupling efficiency increased from 75.8% to 75.92%, with a slope of only 0.04. However, for the output fiber in in the best coupling position, the coupling efficiency increased from 54% to 75% and the slope of the coupling efficiency increased to 21. This represents a great enhancement, by about 500 times.

### 2.4. Spot Optimization

**Figure 6:**
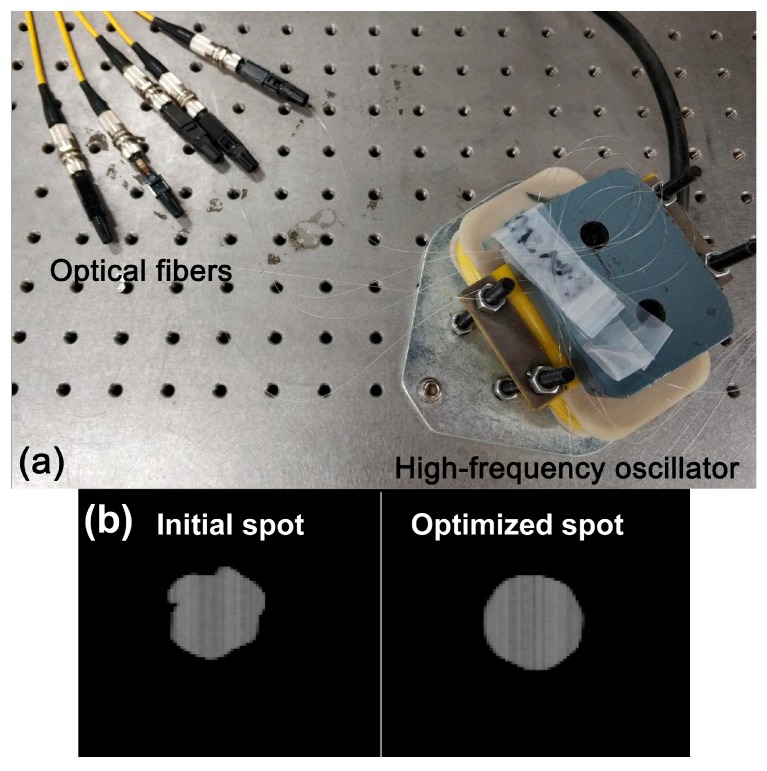
(**a**) Experimental setup of spot optimization; (**b**) the Charged Coupled Device (CCD) images of the initial and optimized spot.

As multimode fiber is adopted as the input in the device, its spot and intensity are random and unstable due to the interference between modes, which leads to unstable test results and large errors. In order to solve this problem, we used a high-frequency oscillator (CZ10, Yong Zhen, China) to produce multi-directional, multi-angle external stress on the multimode fiber, which can cause the output intensity to become stable and uniformly distributed in the spot. The high-frequency oscillator is presented in Figure 6a, on which the optical fibers are crossed and fixed at the cross node. When the oscillator vibrates vertically, the partly fixed bent fibers shake with a certain degree of flexibility, so that multi-directional, multi-angle external stress is generated in the fiber. Because the modes propagating in the fiber are very sensitive to external stress on the fiber, the various kinds of stress will activate a specific mode support by the optical fiber. As the oscillator vibrates with high frequency and the response time of the Charge Coupled Device (CCD) is relatively long, the final image is the accumulation of countless activated modes. These modes tend to be distributed equally, resulting in a spot that exhibits a regular shape and a uniform intensity distribution. Figure 6b compares the images of the output spots in initial and modified states. The black and white stripes vertically lined are caused by the noise of the CCD. In most initial states, the spot appears as a distorted pattern. When external stress is applied, the spot has a regular circular shape, which illustrates that the spot becomes uniform and stable when external force is applied [27,28].

## 3. Fabrication

### 3.1. Process of the Optical Sensor

**Figure 7:**
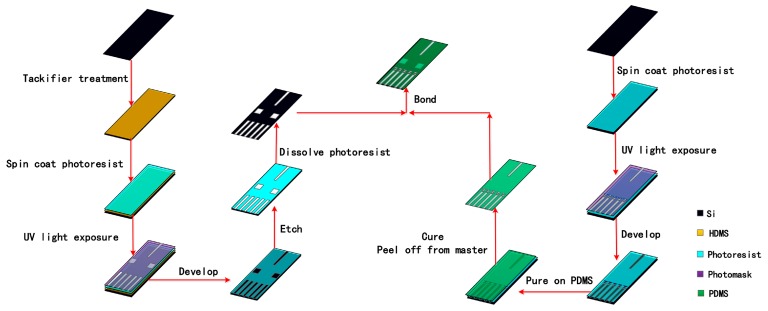
The fabrication process flow of the cell density detector.

**Figure 8:**
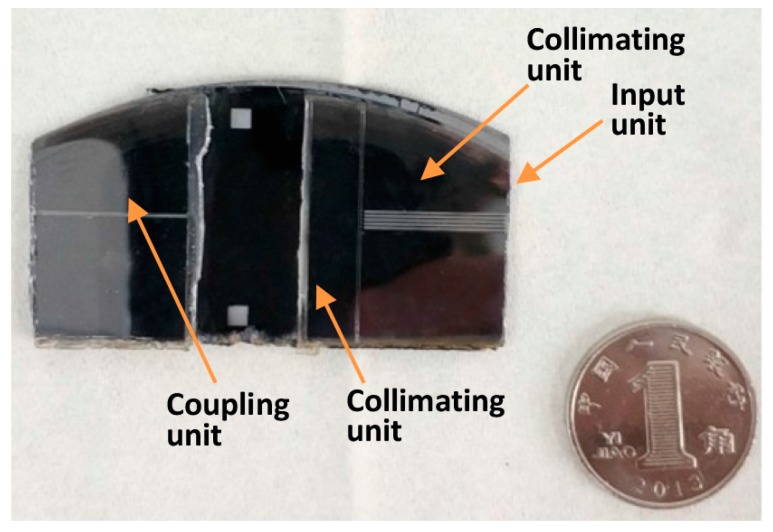
A photograph of the fabricated device together with a Chinese dime.

Figure 7 describes the specific process flow of the designed sensor. The preparation of the optical sensor is mainly divided into two parts: the etching of the designed structure in the silicon substrate and the formation of the polydimethylsiloxane (PDMS) layer. The structure on the silicon substrate was realized by the DIRE (LE0765 LPX DSi, Orbotech, Newport City, UK) and the PDMS layer was prepared by a standard soft lithography process [29,30,31]. After the surface treatment was carried out by an oxygen plasma machine, the prefabricated silicon structure and PDMS layer were bonded together in a bonding machine (EVG 610, EV Group, Schärding, Austria). The surfaces of layers to be bonded were first treated by the oxygen plasma machine. Under the microscope of the bonding machine, the alignment marks formed previously were enlarged, so that the alignment could be realized by carefully adjusting the position and orientation of the PDMS layer. Because the treated surface will lose effectiveness in 3–5 min, the alignment was practiced several times in advance to make sure it could be completed in time. In addition, the thickness of the PDMS layer was made as small as possible (500 μm) to eliminate its deformation during the bonding process. The photograph of the fabricated device together with a dime is shown in Figure 8. In the figure, the detector is about four times the size of a Chinese dime, with a dimension of 45 mm × 25 mm (length × width).

### 3.2. Cell Density Detection System

**Figure 9:**
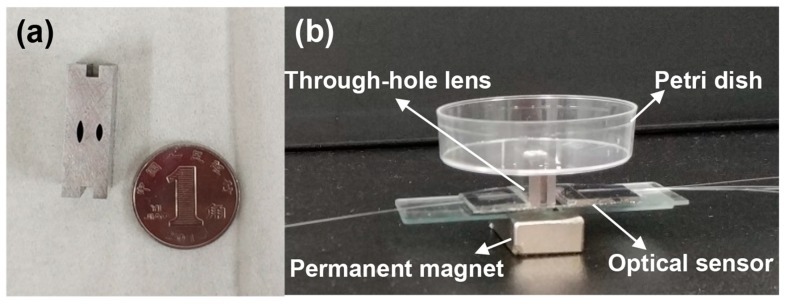
(**a**) Photograph of the container for the cell solution and a Chinese dime; (**b**) Photograph of the cell loading component.

An iron bulk with lens-shaped through-hole was utilized as a container to hold the cell solution (shown in Figure 9a). The square groove is an alignment mark of the focusing lens. In addition, in order to prevent the leakage of cell culture medium, a permanent magnet was used to fix the lens firmly on the surface of the wafer. In order to keep the cells alive during the measurement, a Petri dish was placed on the top of the lens as a container to carry the cell culture fluid. The cell culture medium was composed of 90% DMEM and 10% fetal bovine serum. Figure 9b shows the photograph of the cell loading component with the integrated components.

**Figure 10:**
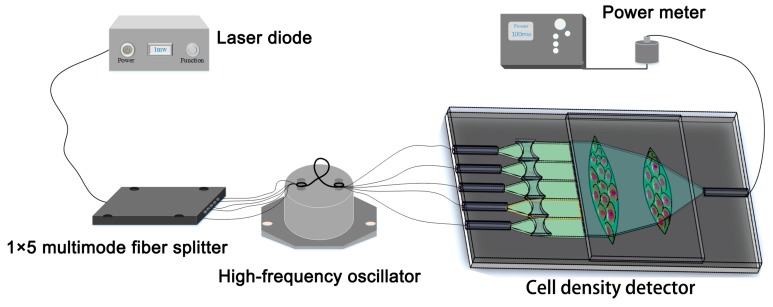
Experimental setup of the cell density test.

The schematic diagram of the experimental setup is shown in Figure 10. Because of the low absorption of the cell in the near-infrared and infrared wavelengths [32], a laser diode (BF-14-DFB1550, wavelength of 1550 nm, My-Aoc Science and Technology, Mianyang, China) was employed as the light source to reduce the influence of the laser thermal effect on the cell physiology. The light emitted from the laser source was divided into five beams by a 1 × 5 multimode fiber splitter. Then, a high-frequency oscillator was employed to achieve the uniform and regular output spot as mentioned above. The light beam passes through the cascaded lenses area, which is attached to the adherent cells. Finally, the collected light intensity was detected by a power meter (1917-R & 918D-IR-OD3R, Newport, Wuxi, China) which varies with the cell density.

## 4. Results and Discussion

**Figure 11:**
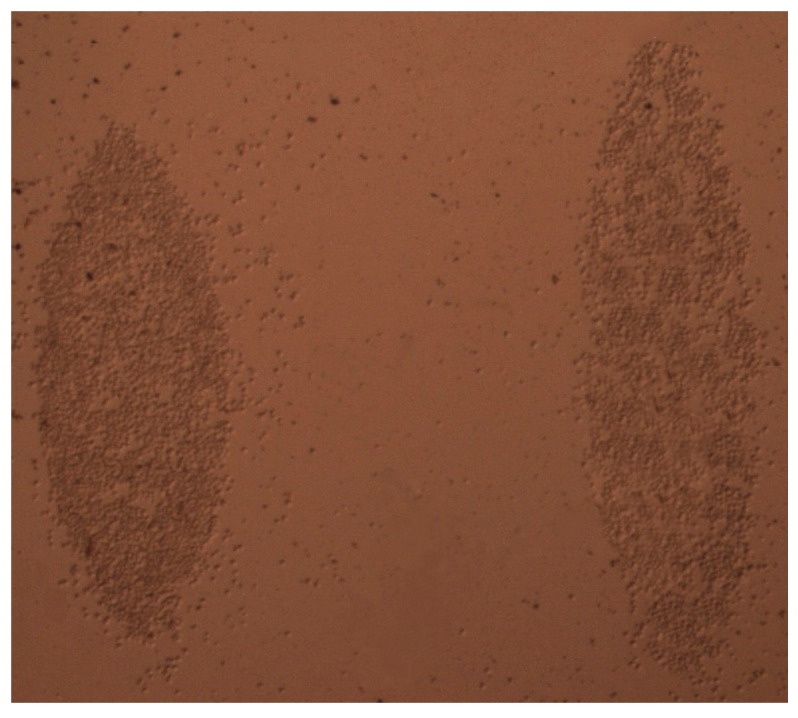
Model diagram of the secondary cascade lens composed of adherent cells.

In the experiment, Hela cells (obtained from the Shanghai Cell Bank of the Chinese Academy, Shanghai, China) were used as an example for the cell density measurement. Firstly, the adherent cells were released from the original culture flask by PBS cleaning, trypsin digestion, and centrifugation. Then, the cells in a suspended state were dripped into the through-hole lens fixed on the fabricated device. In order to promote cell adhesion, the silicon substrate was coated in polylysine. Finally, the optical sensor was placed for a period of time as a pretreatment (2.5 h) to allow the cells to attached evenly to the silicon substrate in the lens range (as shown in Figure 11).

After the pretreatment, the cell loading component was removed, and the focusing unit was tilted and washed slightly by de-ionized (DI) water to purge the residual cell culture solution and possible contaminations. Because the silicon surface is lyophobic, the residual DI water is limited and quickly evaporated in the open environment. As the RI of cell (1.339) was larger than that of its surrounding medium (air, RI = 1), when the cell density in the focusing area (thousands of cells uniformly distributed) changed, the average RI of this area varied.

**Figure 12:**
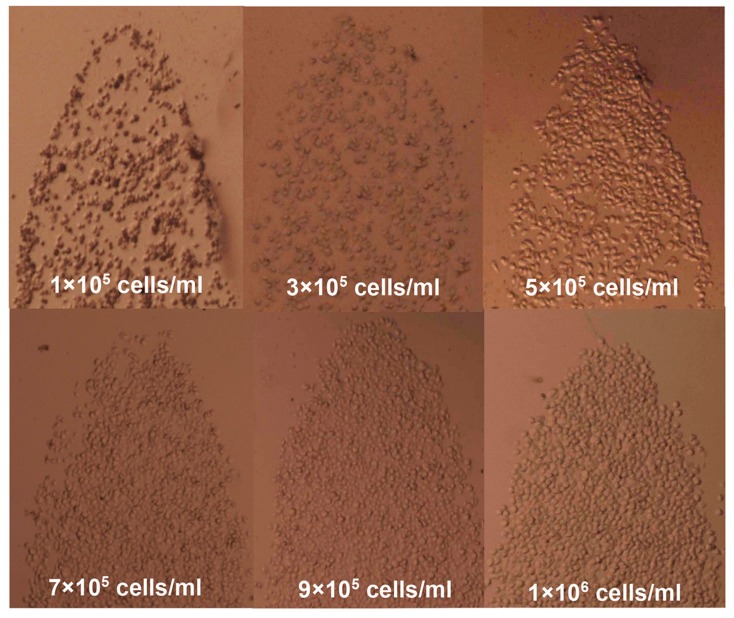
Partial views of the primary lens made up of different Hela cell densities including 1 × 10^5^ cells/mL, 3 × 10^5^ cells/mL, 5 × 10^5^ cells/mL, 7 × 10^5^ cells/mL, 9 × 10^5^ cells/mL, and 1.0 × 10^6^ cells/mL.

Cell solutions with different cell densities (1 × 10^5^ cells/mL, 3 × 10^5^ cells/mL, 5 × 10^5^ cells/mL, 7 × 10^5^ cells/mL, 9 × 10^5^ cells/mL, and 1 × 10^6^ cells/mL) were applied for the density measurement. To clearly illustrate the detailed difference between samples, a series of close-up views of the focusing lenses is provided in Figure 12.

**Figure 13:**
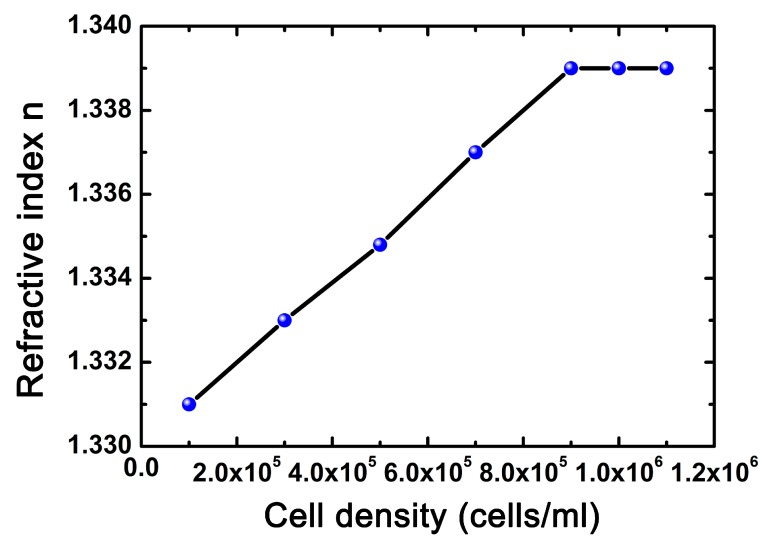
The variation of RI with the change in cell density.

The RI of the cell solutions with different cell densities were measured by a commercial digital RI detector (AR200 digital hand-held refractometer, Reichert Inc., Depew, NY, USA) in advance, and the related result is shown in Figure 13. It can be seen that when the cell density varied between 0.1 × 10^6^ cells/mL and 0.9 × 10^6^ cells/mL, the RI of the cell solution increased almost linearly with the growth of the cell density. Then, when the cell density exceeded 9 × 10^5^ cells/mL, the RI tended to be saturated.

Based on Reference [33], the relationship between the density of the solvent and the RI of the solution can be considered to be approximately linear. In the case of the cell solution, as cells are evenly dispersed in the culture fluid, such a relationship also makes sense. Furthermore, the linear relationship between cell density and RI values has been demonstrated by previous studies [34]. However, some optical parameters of living cells in culture fluid, such as absorption coefficient and extinction coefficient, are still not available, so it is difficult to derive the quantitative relationship between the RI and cell density based on the light–matter interaction [35]. Instead, according to the variation curve obtained by a commercial refractometer (Figure 13), the quantitative relationship was found by data fitting:

(1) $$n=1\times 10^{-8}\cdot c+1.33$$

where *n* and *c* are the RI and cell density of the Hela cell solution, respectively. Here, the unit of cell density *c* is cells/mL.

**Figure 14:**
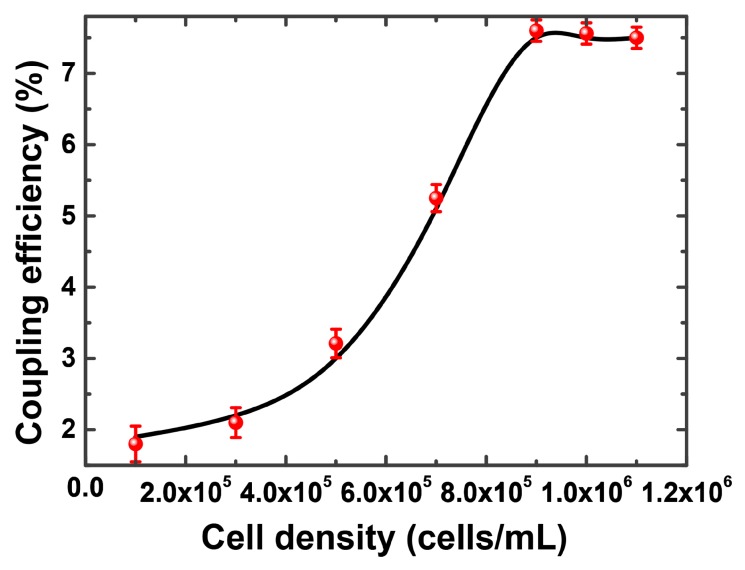
The dependence of the coupling efficiency on the cell density. The measurement was replicated five times, and the error bar represents the standard deviation.

Figure 14 shows the variation of coupling efficiency against cell density. The measurement was replicated five times, and the error bar represents the standard deviation. In the experiment, the coupling efficiency is the efficiency of optical power transfer through the whole device, expressed as the ratio of the input power to the power obtained from the output side. If there are no cells on the chip, the power collected from the output side is particularly small (about 50 nW). When the cell density is within the range of 1 × 10^5^ cells/mL and 1.0 × 10^6^ cells/mL, the coupling efficiency gradually rises from 1.8 to 7.6% (light power from 9 to 38 μW). When the cell density is greater than 1.0 × 10^6^ cells/mL, the coupling efficiency tends to be stable. Therefore, the density of the cell sample obtained by the light intensity coupling efficiency was demonstrated. The sensor sensitivity was calculated to be −76 dB/(cells/mL) by solving the slope of the curve ranging from 3 × 10^5^ to 9 × 10^5^ cells/mL in Figure 14. According to Reference [36,37], the LOD in biological sensors can be express as:

(2) $$\mathrm{LOD}=\frac{3\sigma }{S}$$

where σ is the standard deviation of the total noise of the system and *S* indicates the sensitivity of the sensors.

As the standard deviation for our device was 0.25%, the detection limit of the sensor reached 8.3 × 10^4^ cells/mL. According to its principle, the sensor has the potential to be applied to other kinds of adherent cells (besides Hela cells). The viability and morphological properties of cells affect the performance of the designed sensor. Some other factors, including scattering among cells, dispersion of light, and uniformity in cell density of the two lenses, are considered as error sources for the experimental results.

Due to their biological nature, only the adhered cells with a density larger than a certain value are apt to be attached to the surface, so cells with a density less than the order of 10^5^ cells/mL are not recommended to be measured by the designed sensor. However, a smaller detection range of cell density can be achieved by optimizing the dimensions of the sensing element (e.g., a narrower collimated light beam propagates through the smaller convex lens composed of less adhered cells). For a sample with a cell density higher than the order of 10^6^ cells/mL, it can be diluted to a proper density for measurement and the real value can be obtained by related data processing. Therefore, the design proposed in this study has the potential to achieve a larger measurement range.

It is to be noted that there are some limitations to the designed sensor: (1) this device cannot carry out live/dead analysis for the detected cells; (2) the typical volume of the cell solution utilized for the measurement is 100 μL, which is not suitable for small volume tests; (3) although the focused light intensity can by recorded in several seconds, the time spend on the cell pretreatment (2.5 h) is long for the measurement. All of these limitations provide directions for the further optimization of the sensor.

## 5. Conclusions

In this study, a cell density sensor based on light beam focusing was presented. Theoretical analysis of the sensor structure was carried out based on the TMM and Zemax simulations, and the optimal parameters of the collimating lens and focusing lens were obtained from the simulations, respectively. The high-frequency oscillator was adopted to obtain the output spot from the multimode fiber with a regular shape and uniform intensity distribution. It was demonstrated that the transmission light intensity varies due to the change of the RI of the focusing unit induced by the cell densities. When the cell density changed from 1 × 10^5^ cells/mL to 1 × 10^6^ cells/mL, the light-coupling efficiency rose from 1.9% to 7.5% and the detection limit reached 8.3 × 10^4^ cells/mL. The decrease of coupling efficiency compared to the simulation result was mainly due to processing errors and cell surface scattering. Compared with other conventional methods, the merits and features of our designed sensor are summarized as follows. Due to its low fabrication cost and simple operation, the counting plate is a classic and widely used device/instrument for cell density measurement. However, this device cannot distinguish target cells with contaminations with similar appearance and it has large subjective error. For other two kinds of methods, Coulter counters and flow cytometry, they have high performance concerning cell counting (high accuracy and high throughput), but their prices are also high (thousands of dollars) due to their dedicated structures and complicated signal analysis methods. Compared with the classic detection system of cell density, our cost-effective system can achieve the label-free (no staining) and accurate measurement of cell density for adherent cell types.

## Figures and Tables

**Table 1:** Design parameters of the collimating lens.

Parameters	Symbol	Values
The radius of the collimating lens	*R*	403.55 μm
The spacing of the lens	*L*	200 μm
The thickness of the lens	*T*	125 μm
Refractive index (RI) of polydimethylsiloxane (PDMS)	*n*_PDMS_	1.406
RI of air	*n*_air_	1

**Table 2:** Design parameters of the focusing unit.

	Parameter	Symbol	Values (mm)
The primary lens	Radius	*R*_1_	4
	Width	*D*_1_	1.05
The secondary lens	Radius	*R*_2_	2.5
	Width	*D*_2_	1.05
The distance between two lenses	-	*B*	1
